# CDK11 negatively regulates Wnt/β-catenin signaling in the endosomal compartment by affecting microtubule stability

**DOI:** 10.20892/j.issn.2095-3941.2019.0229

**Published:** 2020-05-15

**Authors:** Danmin Ou, Lin Chen, Jiang He, Zhuoxian Rong, Jie Gao, Zhi Li, Liyu Liu, Feiyu Tang, Jiang Li, Yuezhen Deng, Lunquan Sun

**Affiliations:** ^1^Department of Oncology, Center for Molecular Medicine, Xiangya Hospital, Central South University, Changsha 410008, China; ^2^International Cooperation Base of Cancer Precision Therapy, Department of Science and Technology of Hunan Province, Changsha 410008, China; ^3^Key Laboratory of Molecular Radiation Oncology of Hunan Province, Changsha 410008, China; ^4^National Clinical Research Center for Geriatric Disorders, Changsha 410008, China

**Keywords:** Wnt/β-catenin signaling, CDK11, endosome, microtubule, SIRT2

## Abstract

**Objectives:** Improper activation of Wnt/β-catenin signaling has been implicated in human diseases. Beyond the well-studied glycogen synthase kinase 3β (GSK3β) and casein kinase 1 (CK1), other kinases affecting Wnt/β-catenin signaling remain to be defined.

**Methods:**To identify the kinases that modulate Wnt/β-catenin signaling, we applied a kinase small interfering RNA (siRNA) library screen approach. Luciferase assays, immunoblotting, and real-time polymerase chain reaction (PCR) were performed to confirm the regulation of the Wnt/β-catenin signaling pathway by cyclin-dependent kinase 11 (CDK11) and to investigate the underlying mechanism. Confocal immunofluorescence, coimmunoprecipitation (co-IP), and scratch wound assays were used to demonstrate colocalization, detect protein interactions, and explore the function of CDK11.

**Results:** CDK11 was found to be a significant candidate kinase participating in the negative control of Wnt/β-catenin signaling. Down-regulation of CDK11 led to the accumulation of Wnt/β-catenin signaling receptor complexes, in a manner dependent on intact adenomatosis polyposis coli (APC) protein. Further analysis showed that CDK11 modulation of Wnt/β-catenin signaling engaged the endolysosomal machinery, and CDK11 knockdown enhanced the colocalization of Wnt/β-catenin signaling receptor complexes with early endosomes and decreased colocalization with lysosomes. Mechanistically, CDK11 was found to function in Wnt/β-catenin signaling by regulating microtubule stability. Depletion of CDK11 down-regulated acetyl-α-tubulin. Moreover, co-IP assays demonstrated that CDK11 interacts with the α-tubulin deacetylase SIRT2, whereas SIRT2 down-regulation in CDK11-depleted cells reversed the accumulation of Wnt/β-catenin signaling receptor complexes. CDK11 was found to suppress cell migration through altered Wnt/β-catenin signaling.

**Conclusions:** CDK11 is a negative modulator of Wnt/β-catenin signaling that stabilizes microtubules, thus resulting in the dysregulation of receptor complex trafficking from early endosomes to lysosomes.

## Introduction

Canonical Wnt/β-catenin signaling (denoted Wnt/β-catenin signaling hereafter) is important for embryonic development and organ homeostasis in adults^1^. Abnormal Wnt/β-catenin signaling activity has been reported in many human diseases, such as cancer^1–3^. In the absence of Wnt ligands, β-catenin is phosphorylated, ubiquitinated, and targeted for degradation in a destruction complex consisting of adenomatosis polyposis coli (APC), Axin1, and glycogen synthase kinase 3β (GSK3β) in the cytoplasm^4–6^. The binding of Wnt ligands to Frizzled (Fzd) and low-density lipoprotein receptor-related protein 6 (LRP6) results in recruitment of Dvl2, Axin1, and GSK3β, thus leading to the release of β-catenin. β-catenin then enters the nucleus and activates Wnt/β-catenin signaling^7–9^. LRP6 is phosphorylated after the binding of Wnt ligands to receptors, and Wnt/β-catenin signaling receptor complexes, consisting of Fzd, LRP6, pLRP6, Dvl2, Axin1, and GSK3β, are formed^10–12^. The receptor complexes are internalized into cells through endocytosis^13^ and subjected to endocytic trafficking to lysosomes, where they are degraded^14,15^, thus decreasing signaling activity. An extensive study of epidermal growth factor receptor (EGFR) has indicated that internalized EGFR can signal in early endosomes, whereas signaling terminates in lysosomes, where EGFR is degraded^16^. However, although the endosomal compartment has been implicated as a signaling center^17^, the subcellular location of Wnt/β-catenin signaling receptor complexes remains controversial^12,13^.

Phosphorylation of signaling components by kinases has been shown to regulate various steps of Wnt/β-catenin signaling. Dozens of kinases have been demonstrated to be involved in the phosphorylation of molecules in Wnt/β-catenin signaling cascades^18–25^. However, given the complexity of Wnt/β-catenin signaling, the precise mechanisms regulated by different kinases at different steps remain poorly understood. Thus, an extensive analysis of other kinase functions in Wnt/β-catenin signaling is warranted.

Cyclin-dependent kinase 11 (CDK11) is a serine/threonine kinase encoded by two nearly identical genes, *CDC2L1* and *CDC2L2*^26^. CDK11 is versatile among CDK family members, besides cell cycle and mitosis regulation^27–32^, previous studies have shown that CDK11 is involved in the regulation of transcription and RNA splicing^33–38^, modulation of microtubule stabilization, and autophagy^39,40^. In the present study, we performed a large-scale kinase RNA interference (RNAi) screen to identify kinases that might be involved in Wnt/β-catenin signaling. Among the significant hits, CDK11 was found to be a negative regulator of Wnt/β-catenin signaling. Biochemical and functional analyses demonstrated that CDK11 interacts with SIRT2 in modulating tubulin stability, thereby affecting the cellular trafficking of Wnt/β-catenin signaling receptor complexes from early endosomes to lysosomes. Our findings suggest a new mechanism whereby Wnt/β-catenin signaling is negatively regulated.

## Materials and methods

### Cell lines, plasmids, antibodies, and reagents

HeLa, HCT116, and SW480 cells were maintained in RPMI 1640 (Hyclone, Logan, UT, USA), and HEK293T cells were maintained in DMEM (Hyclone) supplemented with 10% fetal bovine serum (FBS) (Gibco, Carlsbad, CA, USA). The TOPFlash luciferase reporter plasmid was purchased from Addgene (Watertown, MA, USA), the pRL-TK *Renilla* luciferase reporter plasmid was purchased from Promega (Madison, WI, USA), and the CDK11-Flag plasmid was purchased from Genscript (Nanjing, China). Wnt3a was purchased from R&D Systems (Minneapolis, MN, USA). Antibodies to the following were used: β-catenin, CDK11, and MEC-17 (Abcam, Cambridge, MA, USA); LRP6, pLRP6, Axin1, phospho-β-catenin (Ser33/Ser37/Thr41), GSK3β, acetyl-α-tubulin, histone deacetylase 6 (HDAC6), tubulin, and N-cadherin (CST, Danvers, MA, USA); Dvl2, EEA1, and LAMP1 (Santa Cruz Biotechnology, Santa Cruz, CA, USA); Flag, glyceraldehyde-3-phosphate dehydrogenase (GAPDH), and SIRT2 (Sigma-Aldrich, St. Louis, MO, USA); and TSG101 (GeneTex, Irvine, CA, USA). In addition, normal rabbit IgG, horseradish peroxidase (HRP)-conjugated secondary mouse antibody, and HRP-conjugated secondary rabbit antibody (CST) were used.

### Kinase RNAi library, small interfering RNA (siRNA), and plasmid transfection

The siGENOME SMARTpool Library-Human Protein Kinase was purchased from Dharmacon (Cambridge, MA, USA). Cotransfection of the kinase siRNA library and luciferase reporter plasmids (at a 200:1 ratio of TOPFlash plasmid to pRL-TK plasmid in micrograms) followed the protocol of DharmaFECT® Duo Transfection Reagent (Thermo Scientific, Waltham, MA, USA).

The siRNAs targeting various genes were purchased from GenePharma (Suzhou, China), and their sequences were as follows (sense strand 5′-3′): β-catenin: AGGUGCUAUCUGUCUGCUC; CDK11-1: AUGGAGUGGUCUACAGAGCAA; CDK11-2: AGAUCU ACAUCGUGAUGAA; HRS: CGUCUUUCCAGAAUUCAAA; TSG101: CAGUUUAUCAUUCAAGUGUAA; EAP20: CGAUCCAGAUUGUAUUAGA; CHMP6: AGAUCGAAA UGAAAGUGAU; LRP6: CCAUGGAUAUACAUGCUUU; and SIRT2: CAGCGCGUUUCUUCUCCUGUA. siRNA transfection was performed according to the protocol of DharmaFECT transfection reagent (Dharmacon).

Plasmid transfection was carried out with ViaFect™ transfection reagent (Promega) according to the manufacturer’s instructions.

### Luciferase assay

Luciferase activity was measured with the Dual-Glo® Luciferase Assay System (Promega) according to the manufacturer’s protocol. In brief, Duo-Glo® luciferase reagent was added to cells (75 μL/well) that had been grown in 96-well plates with complete medium. Firefly luminescence was measured after incubation at room temperature for 20 min, and equal amounts of Duo-Glo® Stop & Glo® reagent (75 μL/well) were added to the plates, which were further incubated for 20 min at room temperature. Subsequently, *Renilla* luciferase luminescence was measured. The ratio of firefly to *Renilla* luciferase luminescence for each well was calculated as the relative luciferase activity.

### Real-time polymerase chain reaction (PCR)

Cells were lysed in TRIzol reagent (Invitrogen, Carlsbad, CA, USA), and total RNA was extracted according to the manufacturer’s protocol. RNA was reverse transcribed into cDNA with a kit (TaKaRa, Dalian, China), and SYBR Green-based real-time PCR was performed to quantify mRNA expression according to the manufacturer’s protocol (Bio-Rad, Hercules, CA, USA). Relative mRNA expression was normalized to GAPDH expression. The primer sequences were as follows (5′-3′): c-myc (forward: CCTGGTGCTCCATGAGGAGAC and reverse: CAGACTCTGACCTTTTGCCAGG), Axin2 (forward: CAAACTTTCGCCAACCGTGGTTG and reverse: GGTGCAAAGACATAGCCAGAACC), LRP6 (forward: CAGTTGGAGTGGTGCTGAAAGG and reverse: CCATCCAAAGCAGCCCGTTCAA), Dvl2 (forward: TCCATACGGACATGGCATCGGT and reverse: CGTGATGGTAGAGCCAGTCAAC), Axin1 (forward: GTATGTGCAGGAGGTTATGCGG and reverse: CACCTTCCTCTGCGATCTTGTC), GSK3β (forward: CCGACTAACACCACTGGAAGCT and reverse: AGGATGGTAGCCAGAGGTGGAT), CDK11 (forward: CCGACTTACAGGACATCAGCGA and reverse: CTCCTCTGATTCTTCACTGGTGC), EEA1 (forward: CTTCTAGCCACCAGGCAAGATC and reverse: CCAATGTAGCCTTGGCAGTCTTC), LAMP1 (forward: CGTGTCACGAAGGCGTTTTCAG and reverse: CTGTTCTCGTCCAGCAGACACT), HRS (forward: GACAGACTCTCAGCCCATTCCT and reverse: TCATGCGGTTCACGAAGGTGGT), TSG101 (forward: TTCTCAGCCTCCTGTGACCACT and reverse: CCATTTCCTCCTTCATCCGCCA), EAP20 (forward: AGAGCAAGTCCAGCTTCCTGATC and reverse: GGTAAAGACGGAGTTGTTCTGGC), and CHMP6 (forward: GACAAGCTGAGGCAGTACCAGA and reverse: CTGCTCCTGGTATCGCTTCTTC).

### Immunoblotting

Cells were washed 3 times with cold phosphate-buffered saline (PBS) and lysed on ice with lysis buffer [50 mM Tris hydrochloride (HCl), 150 mM sodium chloride (NaCl), 0.5% Triton X-100 (Sigma-Aldrich), 2 mM ethylenediaminetetraacetic acid (EDTA) (pH 8.0)] supplemented with 1% protease inhibitor (Bimake, Houston, TX, USA) and 1% phosphatase inhibitor (Bimake) for 30 min. Cell debris was removed by centrifugation at 14,000 *g* for 15 min at 4 °C. Protein concentrations were measured with a BCA protein assay kit (Thermo Scientific) according to the manufacturer’s protocol. Twenty micrograms of protein for each sample was separated on 6%–10% polyacrylamide gels after denaturation for 5 min and transferred to polyvinylidene fluoride membranes (Millipore, Burlington, MA, USA). After being blocked with 5% nonfat milk in Tris-buffered saline containing Tween 20 (TBST; 1:1000) for 1 h at room temperature, the membranes were incubated with primary antibody overnight at 4 °C. After being washed with TBST 3 times for 15 min, membranes were incubated with HRP-conjugated secondary antibody for 1 h at room temperature. Primary and secondary antibodies were diluted with 5% nonfat milk in TBST. Super Enhanced Chemiluminescence Substrate (Millipore) was used to detect proteins after membranes were washed with TBST 3 times for 15 min.

### Immunofluorescence

Cells grown on coverslips were rinsed 3 times in PBS, fixed in fresh 4% (w/v) paraformaldehyde (Sigma-Aldrich) in PBS for 15 min, rinsed another 3 times in PBS, and then permeabilized and blocked with 0.3% Triton X-100 and 5% bovine serum albumin (BSA) in PBS for 30 min at room temperature. The cells were then incubated overnight with primary antibodies diluted in 0.15% Triton X-100 and 2.5% BSA in PBS at 4 °C. After being washed with 0.1% Triton X-100 in PBS for 10 min and rinsed twice in PBS, the cells were incubated for 1 h at 37 °C with Alexa Fluor® 488-conjugated secondary antibody (Invitrogen) or Alexa Fluor® 594-conjugated secondary antibody (Invitrogen) diluted in 0.15% Triton X-100 and 2.5% BSA in PBS; the cells were then washed with 0.1% Triton X-100 in PBS for 10 min and rinsed twice in PBS. DNA was stained for 5 min with 1 μg/mL 4′,6-diamidino-2-phenylindole (CST) diluted in PBS. Images were taken with a confocal fluorescence microscope.

### Coimmunoprecipitation (co-IP) assay

Protein preparation and quantification were performed as described above. Immunoprecipitation experiments were performed with protein A/G magnetic beads (Bimake) according to the manufacturer’s protocol. In brief, magnetic beads were precleared with lysis buffer and incubated with equal amounts of anti-CDK11 and normal rabbit IgG diluted in 50 μL lysis buffer, for 4 h at 4 °C in a rotator. Then, the beads were washed twice with 150 μL lysis buffer, and 2 mg protein was added to the magnetic beads and incubated for another hour at 4 °C in the rotator. The beads were washed four times with 300 μL lysis buffer and then resuspended in 50 μL protein loading buffer and denatured for 5 min by boiling. Protein analysis was performed by immunoblotting.

### Scratch wound assays

Cells were seeded in 12-well plates. A scratch was made with a 10 μL pipette tip after cells had reached approximately 90% confluence. PBS was used to wash the cells 3 times to remove floating cells. Serum-free medium was then applied to the cell culture. Images were taken at 0 and 48 h after the scratch assay was performed.

### Statistical analysis

All data are presented as mean ± standard deviation (SD). The differences between two groups were analyzed with Student’s* t*-test, and the differences among 3 or more groups were analyzed with one-way analysis of variance (ANOVA). A Chi-squared (χ^2^) test was used to analyze the immunofluorescence overlap ratio. The significance level was set at *P* < 0.05.

## Results

### RNAi screen for candidate kinases regulating Wnt/*β*-catenin signaling

**Figure 1:**
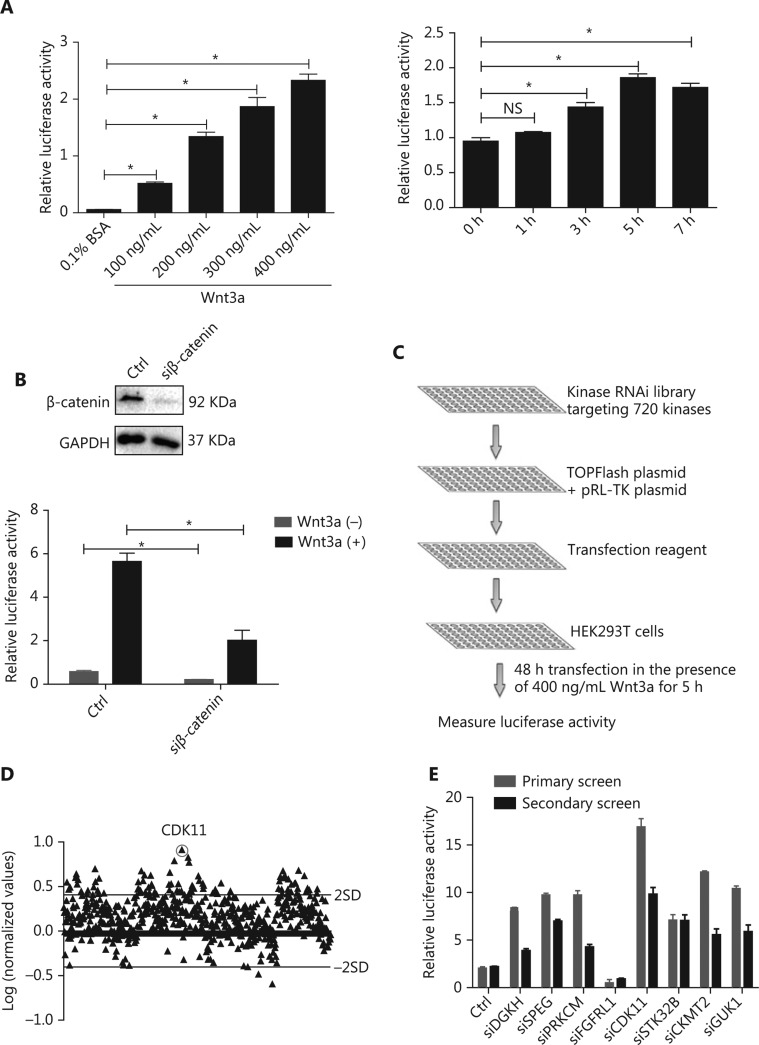
RNAi screen of kinases regulating Wnt/β-catenin signaling activity. (A) Relative luciferase activity in HEK293T cells cotransfected with TOPFlash and pRL-TK plasmids under Wnt3a stimulation at different doses and time points (*P < 0.05; NS, no statistical significance). (B) Relative luciferase activity in HEK293T cells cotransfected with luciferase reporter plasmids and nontargeting siRNA or β-catenin siRNA with or without Wnt3a stimulation. Top, results of β-catenin depletion (*P < 0.05). (C) Schematic of the RNAi screen strategy. (D) Log ratio of the relative luciferase activity of targeting kinase siRNAs to nontargeting siRNAs, under Wnt3a stimulation, in HEK293T cells. Two SD of the log ratios of 720 candidates were quantified and are indicated in the figure. (E) Secondary screen for Wnt/β-catenin signaling activity of 8 representative kinases in HEK293T cells was performed and is shown with the primary screen results. All results are representative of 3 independent experiments.

To identify kinases involved in Wnt/β-catenin signaling, we designed an approach to recapitulate Wnt/β-catenin signaling. In this assay, recombinant Wnt3a was used to activate Wnt/β-catenin signaling in HEK293T cells cotransfected with TOPFlash plasmid, a classical reporter plasmid for Wnt/β-catenin signaling, and pRL-TK plasmid, an internal control. The ratio of TOPFlash luciferase activity normalized to pRL-TK luciferase activity represented the relative activity of Wnt/β-catenin signaling. To determine the optimized concentration and time of Wnt3a stimulation, we tested different doses and time points of Wnt3a stimulation. Wnt3a strongly increased Wnt/β-catenin signaling activity in a dose- and time-dependent manner (*P* < 0.05; **Figure 1A**). The optimal conditions for Wnt/β-catenin signaling activity in HEK293T cells were 400 ng/mL Wnt3a stimulation for 5 h. To further optimize the transfection time for the large-scale RNAi screen, we used siRNA against β-catenin as a positive control and compared the response of HEK293T cells cotransfected with luciferase reporter plasmids plus nontargeting siRNA or siRNA against β-catenin with or without Wnt3a treatment. The system was shown to be sensitive to β-catenin depletion after 48 h of transfection (*P* < 0.05; **Figure 1B**). Subsequently, a large-scale RNAi screen was performed after the siRNA library was cotransfected with luciferase reporter plasmids for 48 h with 400 ng/mL Wnt3a stimulation for 5 h.

Under the above optimized conditions, we conducted kinase library screen with a library targeting 720 genes encoding kinases (4 siRNAs per gene as a pool) (**Figure 1C**). To identify kinases involved in Wnt/β-catenin signaling, we determined the ratio of the relative luciferase activity of the targeting siRNA group to that of the nontargeting siRNA group, both of which received Wnt3a treatment, and converted the results to logarithm scale. Only the kinases that increased or decreased Wnt/β-catenin signaling activity by 2 SD for all siRNAs of the library were considered potentially positive hits (**Figure 1D**). To verify the effectiveness of the primary screen, we chose 8 candidates for secondary screen to confirm their effects on Wnt/β-catenin signaling by the repeating luciferase activity measurements. The results were consistent with those after the first-round screen (**Figure 1E**). Among the positive hits, CDK11 was one of the most effective and reproducible kinases found to regulate Wnt/β-catenin signaling (**Figure 1D and 1E**).

### CDK11 negatively regulates Wnt/*β*-catenin signaling

**Figure 2:**
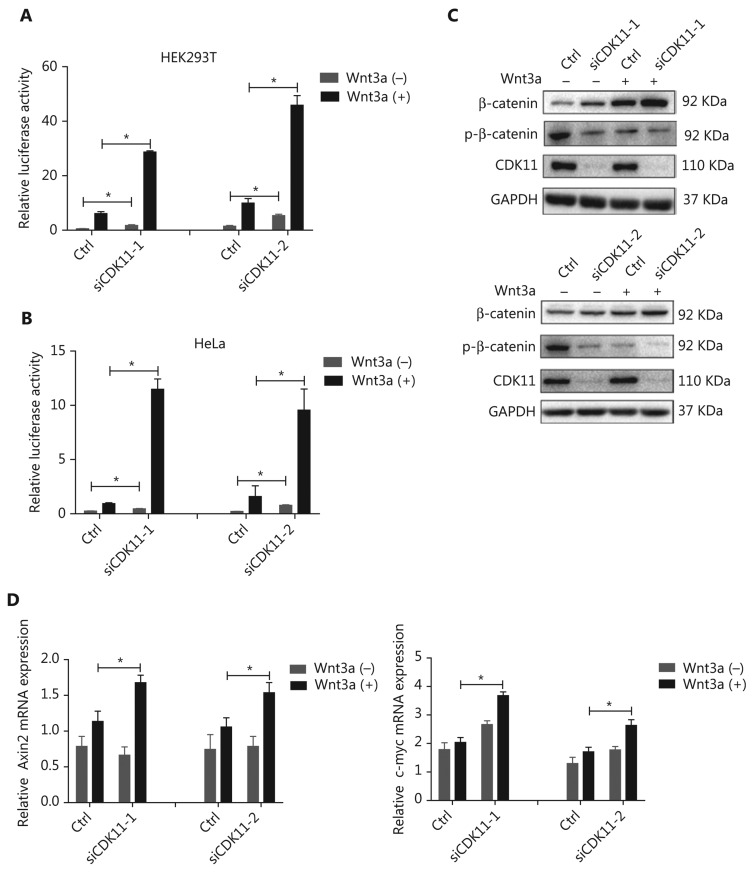
Negative regulation of Wnt/β-catenin signaling by CDK11. (A) Relative luciferase activity in CDK11-depleted HEK293T cells with or without Wnt3a stimulation (*P < 0.05). (B) Relative luciferase activity in CDK11-depleted HeLa cells with or without Wnt3a stimulation (*P < 0.05). (C) Immunoblotting analysis of β-catenin and phospho-β-catenin (Ser33/Ser37/Thr41) expression in CDK11-depleted HeLa cells with or without Wnt3a stimulation. (D) mRNA levels of Axin2 and c-myc measured by real-time polymerase chain reaction (PCR) in CDK11-depleted HeLa cells with or without Wnt3a stimulation (*P < 0.05). All results are representative of 3 independent experiments.

To confirm the modulation of CDK11 in Wnt/β-catenin signaling and eliminate possible off-target effects, we performed a CDK11 loss-of-function assay with 2 newly synthesized siRNAs whose target sequences were completely different from that of CDK11 in the kinase RNAi library. Knockdown of CDK11 significantly increased Wnt/β-catenin signaling activity in HEK293T cells (*P* < 0.05; **Figure 2A**). Because Wnt/β-catenin signaling activity in untreated HEK293T cells is at a basal level, HeLa cells in which Wnt/β-catenin signaling was active were used in the rest of the assays unless otherwise indicated. As shown in the luciferase assay results, depletion of CDK11 also increased Wnt/β-catenin signaling activity in HeLa cells (*P* < 0.05; **Figure 2B**). In addition, knockdown of CDK11 increased the expression of total β-catenin and decreased the expression of phospho-β-catenin (**Figure 2C**), a hallmark of Wnt/β-catenin signaling activation. Furthermore, we tested the effect of CDK11 depletion on the expression of the downstream target genes of Wnt/β-catenin signaling and found that the mRNA levels of Axin2 and c-myc were up-regulated under Wnt3a stimulation (*P* < 0.05; **Figure 2D**). Thus, our data indicate that CDK11 is a negative regulator of Wnt/β-catenin signaling.

### CDK11 is involved in modulating receptor complexes of Wnt/*β*-catenin signaling

**Figure 3:**
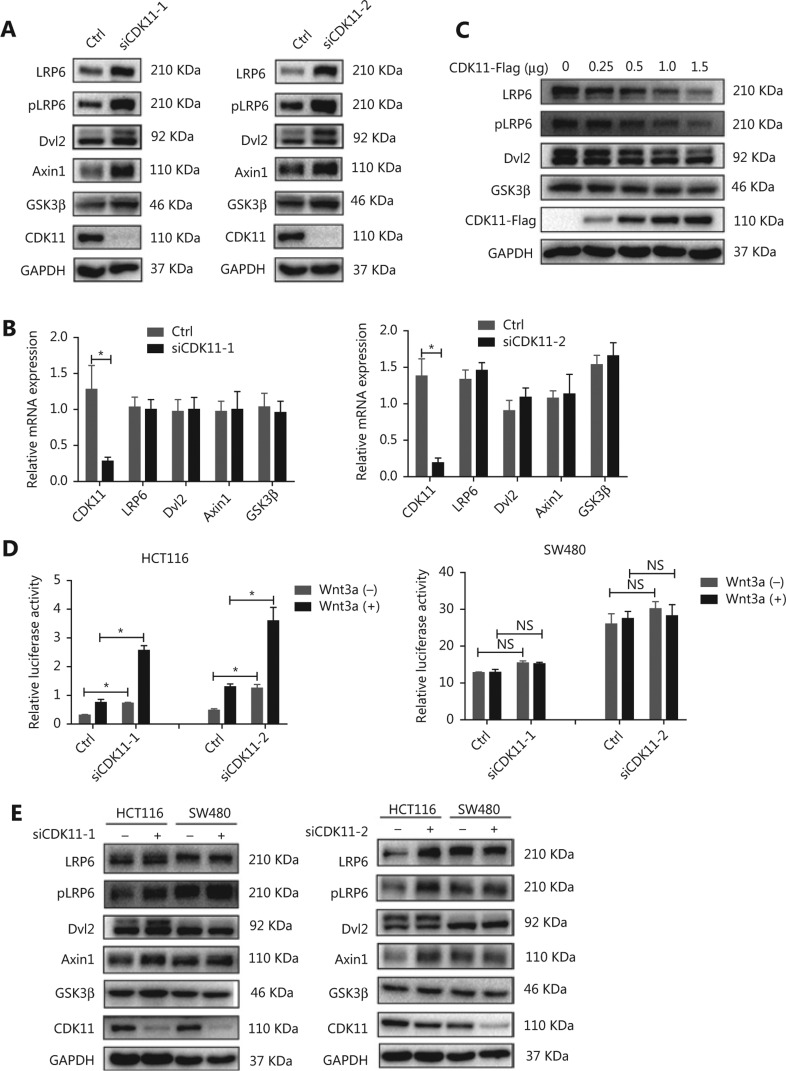
Wnt/β-catenin signaling components accumulate after CDK11 depletion in an intact APC-dependent manner. (A) Western blot analysis of LRP6, pLRP6, Dvl2, Axin1, and GSK3β expression in CDK11-depleted HeLa cells. (B) mRNA levels of LRP6, Dvl2, Axin1, and GSK3β, measured by real-time PCR in CDK11-depleted HeLa cells (*P < 0.05). (C) Western blot analysis of LRP6, pLRP6, Dvl2, and GSK3β expression in CDK11-overexpressing HeLa cells. (D) Relative luciferase activity in CDK11-depleted HCT116 and SW480 cells with or without Wnt3a stimulation (*P < 0.05; NS, no statistical significance). (E) Western blot analysis of LRP6, pLRP6, Dvl2, Axin1, and GSK3β expression in CDK11-depleted HCT116 and SW480 cells. All results are representative of 3 independent experiments.

To investigate the mechanism through which CDK11 depletion enhanced Wnt/β-catenin signaling, we assayed the expression of Wnt/β-catenin signaling cascade components after CDK11 depletion. Interestingly, among the signaling cascade components, the protein levels of LRP6, pLRP6, Dvl2, Axin1, and GSK3β, which are components of receptor complexes, were all significantly up-regulated in CDK11-depleted cells (**Figure 3A**), whereas the mRNA levels of LRP6, Dvl2, Axin1, and GSK3β did not change (**Figure 3B**). When CDK11 was overexpressed, LRP6, pLRP6, Dvl2, and GSK3β decreased in a dose-dependent manner (**Figure 3C**), thus further confirming that CDK11 expression affects the protein levels of Wnt/β-catenin signaling receptor complexes.

In the Wnt/β-catenin signaling destruction complex, the tumor suppressor APC is an essential component. To explore whether APC might participate in the regulation of Wnt/β-catenin signaling after CDK11 depletion, we tested the effect of CDK11 depletion on Wnt/β-catenin signaling in two colorectal cancer cell lines: SW480 cells, whose APC was truncated, and HCT116 cells, whose APC was intact. Intriguingly, CDK11 depletion had no effect on Wnt/β-catenin signaling activity in SW480 cells, but Wnt/β-catenin activity was significantly enhanced in HCT116 cells (*P* < 0.05; **Figure 3D**). When we measured the main components of the receptor complexes in those two cells, we found that down-regulation of CDK11 led to increased levels of LRP6, pLRP6, Dvl2, Axin1, and GSK3β in HCT116 cells, but we observed no changes in SW480 cells (**Figure 3E**). These data suggest that CDK11 is involved in the regulation of receptor complexes of Wnt/β-catenin signaling in a manner depended on intact APC.

### CDK11 regulates Wnt/*β*-catenin signaling in endosome-lysosome vacuoles

**Figure 4:**
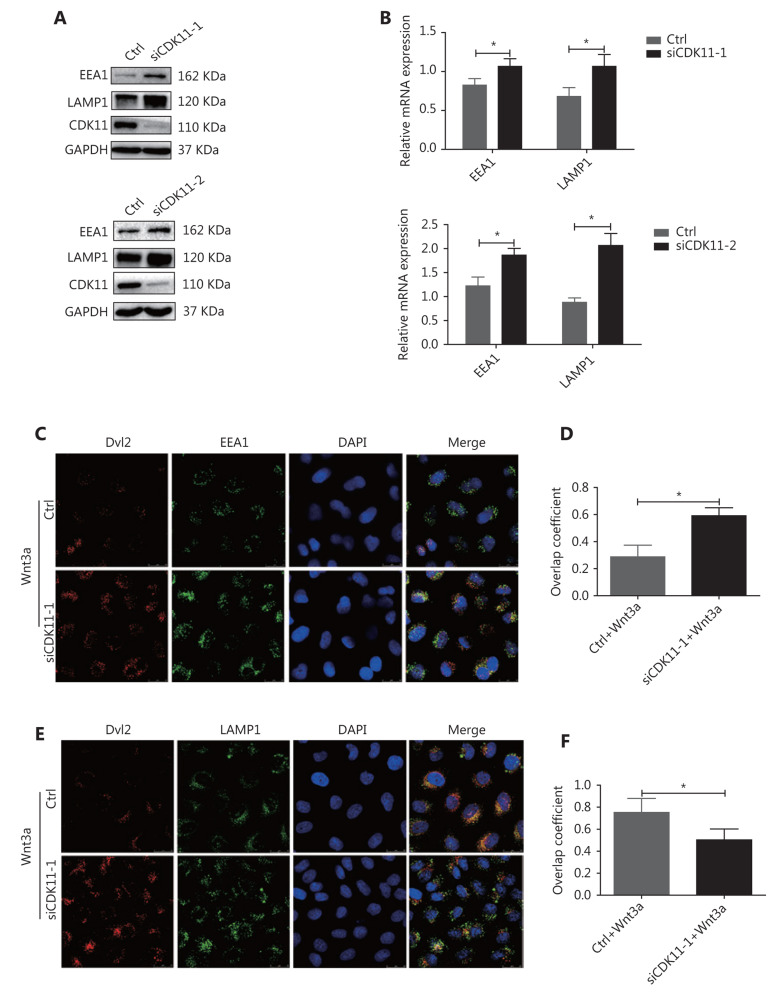
Receptor complexes are stuck in early endosomes in CDK11-depleted cells. (A) Western blot analysis was performed to examine the expression of EEA1 and LAMP1 in CDK11-depleted HeLa cells. (B) Real-time PCR was performed to examine the mRNA levels of EEA1 and LAMP1 in CDK11-depleted HeLa cells (*P < 0.05). (C) In control and CDK11-depleted HeLa cells stimulated with Wnt3a, confocal immunofluorescence assays showed the colocalization of Dvl2 with EEA1 (400×). (D) Quantification of the overlap coefficient of Dvl2 with EEA1 in control and CDK11-depleted cells stimulated with Wnt3a (*P < 0.05). (E) In control and CDK11-depleted HeLa cells stimulated with Wnt3a, confocal immunofluorescence assays showed the colocalization of Dvl2 with LAMP1 (400×). (F) Quantification of the overlap coefficient of Dvl2 with LAMP1 in control and CDK11-depleted cells stimulated with Wnt3a (*P < 0.05). All results are representative of 3 independent experiments.

When Wnts bind their receptors, the receptor complexes are internalized, trafficked, and degraded through the endolysosomal system^13–15^. Because CDK11 was negatively correlated with the protein levels of receptor complexes, and depletion of CDK11 enhanced signaling, we speculated that CDK11 might play a part in the destabilization of Wnt/β-catenin signaling receptor complexes via endolysosomal degradation. To verify this possibility, we first examined the effect of CDK11 depletion on the dynamic changes in the endolysosomal system. As shown in **Figure 4A and 4B**, knockdown of CDK11 significantly increased the expression of the early endosome marker EEA1 and lysosome marker LAMP1 at both the protein and RNA levels (*P* < 0.05). A confocal immunofluorescence assay showed that Dvl2 accumulated more in early endosomes in CDK11-depleted cells stimulated with Wnt3a (**Figure 4C**) but less in lysosomes (**Figure 4E**). **Figure 4D** and **4F** shows the overlap coefficient of Dvl2 with EEA1 and LAMP1, respectively, in CDK11-depleted cells under Wnt3a stimulation (*P* < 0.05). Thus, our data suggest that CDK11 depletion enhances Wnt/β-catenin signaling by retaining the receptor complexes in early endosomes, where Wnt/β-catenin signaling is maintained.

### CDK11 modulation of Wnt/*β*-catenin signaling involves microtubule stability and depends on the endosomal sorting complex required for transport (ESCRT)

**Figure 5:**
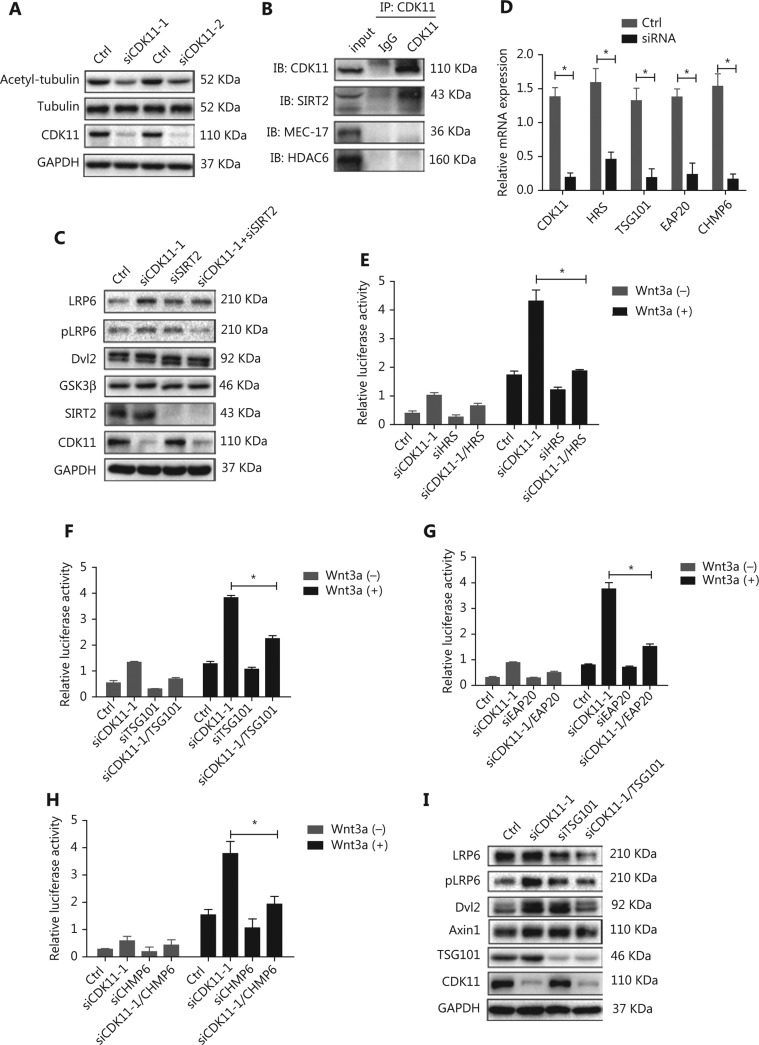
Disruption of microtubule stability and ESCRT reverses the modulation of CDK11 on Wnt/β-catenin signaling. (A) Western blot analysis of acetyl-α-tubulin expression in CDK11-depleted HeLa cells. (B) Co-IP assays were performed to examine the interactions among CDK11 and SIRT2, HDAC6, and MEC-17 in HeLa cells. (C) Western blot analysis of the receptor complex levels of Wnt/β-catenin signaling in CDK11- and SIRT2-depleted HeLa cells. (D) Real-time PCR was performed to examine the expression of CDK11, HRS, TSG101, EAP20, and CHMP6 after knockdown of CDK11, HRS, TSG101, EAP20, and CHMP6, respectively, in HeLa cells (*P < 0.05). (E-H) Luciferase reporter assays showing the effect of ESCRT complex depletion on the activation of Wnt/β-catenin signaling induced by CDK11 depletion in HeLa cells (*P < 0.05). (I) Protein levels of LRP6, pLRP6, Dvl2, and Axin1 were examined in CDK11- and TSG101-depleted HeLa cells. All results are representative of 3 independent experiments.

CDK11 has been suggested to be involved in microtubule stability^39^, which in turn is required for transport from early endosomes to late endosomes or lysosomes^41,42^. We therefore wondered whether CDK11 may play a role in the intracellular trafficking of the receptor complexes between early endosomes and lysosomes via modulation of microtubule stability. First, we found that the expression of acetyl-α-tubulin, a marker of microtubule stability, decreased in CDK11-depleted cells, whereas total tubulin did not change (**Figure 5A**). Further analysis of protein-protein interactions showed that CDK11 interacted with tubulin deacetylase SIRT2 but not with two other well-known enzymes, the tubulin deacetylase HDAC6 and the tubulin acetylase MEC-17 (**Figure 5B**). Knockdown of SIRT2 reversed the expression of receptor complexes of Wnt/β-catenin signaling in CDK11-depleted cells (**Figure 5C**). These data suggest that CDK11 may affect SIRT2 activity and consequently regulate tubulin stability, thereby playing a role in receptor complex trafficking of Wnt/β-catenin signaling.

The ESCRT complex comprises four distinct components (ESCRT-0, ESCRT-I, ESCRT-II, and ESCRT-III) and has been reported to be involved in endosomal sorting^43^. To test whether CDK11 modulation of Wnt/β-catenin signaling might require an ESCRT-mediated mechanism, we knocked down HRS (ESCRT-0), TSG101 (ESCRT-I), EAP20 (ESCRT-II), and CHMP6 (ESCRT-III) individually in CDK11-depleted cells (*P* < 0.05; **Figure 5D**). As shown in **Figure 5E to 5H**, whereas CDK11 knockdown markedly increased the relative luciferase activity, the suppression of HRS, TSG101, EAP20, or CHMP6 significantly reversed the activity in CDK11-depleted cells (*P* < 0.05). Furthermore, Western blot analysis showed that expression of LRP6, pLRP6, Dvl2, and Axin1 decreased after TSG101 was perturbed in CDK11-depleted cells (**Figure 5I**), thus suggesting that the negative regulation of CDK11 on Wnt/β-catenin signaling is dependent on the ESCRT machinery.

### CDK11 affects migration through Wnt/*β*-catenin signaling

**Figure 6:**
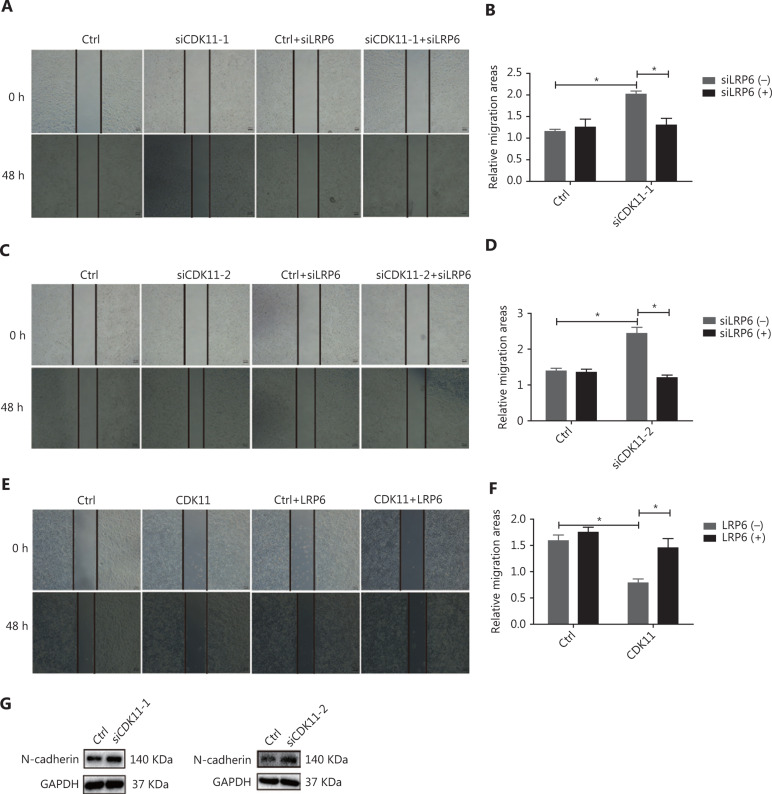
CDK11 depletion promotes migration through Wnt/β-catenin signaling. (A-D) Representative images (50×; A and C) and quantification (B and D) of the scratch wound assay results in CDK11- and LRP6-depleted HeLa cells (*P < 0.05). (E and F) Representative images (50×; E) and quantification (F) of the scratch wound assay results in CDK11- and LRP6-overexpressing HeLa cells (*P < 0.05). (G) Western blot analysis of N-cadherin expression in CDK11-depleted HeLa cells. All results are representative of 3 independent experiments.

To explore the role of CDK11 in cell biology through modulation of Wnt/β-catenin signaling, we performed scratch wound assays, which indicated that the closure of wound gaps increased in CDK11-depleted cells, but down-regulation of LRP6 reversed the modulation (*P* < 0.05; **Figure 6A-D**). The opposite effects were observed in CDK11-overexpressing cells, and overexpression of LRP6 reversed the modulation (*P* < 0.05; **Figure 6E and 6F**). We further analyzed the expression of N-cadherin, which is associated with cell migration; in agreement with the results of our scratch wound assays, N-cadherin was up-regulated in CDK11-depleted cells (**Figure 6G**). The above data suggest that CDK11 plays a role in cell migration through the regulation of Wnt/β-catenin signaling.

## Discussion

Wnt/β-catenin signaling has been implicated in many physiological and pathological processes. Whereas the ligand-mediated activation of Wnt/β-catenin signaling has been well documented, the negative regulation of signaling remains to be elucidated. The key negative regulators described to date have mainly been secreted proteins that antagonize the ligand, such as secreted Frizzled-related proteins and Wnt inhibitory protein (WIF-1), both of which can bind Wnts, thereby inhibiting interactions between Wnts and Wnt receptors^44,45^. Other Wnt inhibitors include Dickkopf-1, which antagonizes signaling by binding LRP5/6^46^. In the present study, we identified CDK11 as a novel negative regulator of Wnt/β-catenin signaling. CDK11 was found to participate in receptor complex trafficking, and down-regulation of CDK11 significantly increased the levels of the receptor complexes, which showed increased accumulation in early endosomes and decreased accumulation in lysosomes, thus enhancing Wnt/β-catenin signaling.

A key question regarding Wnt/β-catenin signaling is the location where the signaling of receptor complexes occurs in cells. A previous study has demonstrated that receptor complexes as a whole can signal but do not colocalize with early endosomes^12^, whereas other studies have suggested that other receptors in early endosomes can signal^17^. Here, in CDK11-depleted cells, the receptor complexes of Wnt/β-catenin signaling accumulated in early endosomes and signaled persistently. This result is probably due to either concentration of active receptor complexes in endosomes or sequestration of GSK3β into endosomes, thus allowing more β-catenin to enter the nucleus and subsequently activate Wnt/β-catenin signaling^47,48^. Although the inadequacy of current technology precludes accurate measurement of signaling from endosomes, our data suggest a role of CDK11 in the negative regulation of Wnt/β-catenin signaling in the endosomal compartment.

**Figure 7:**
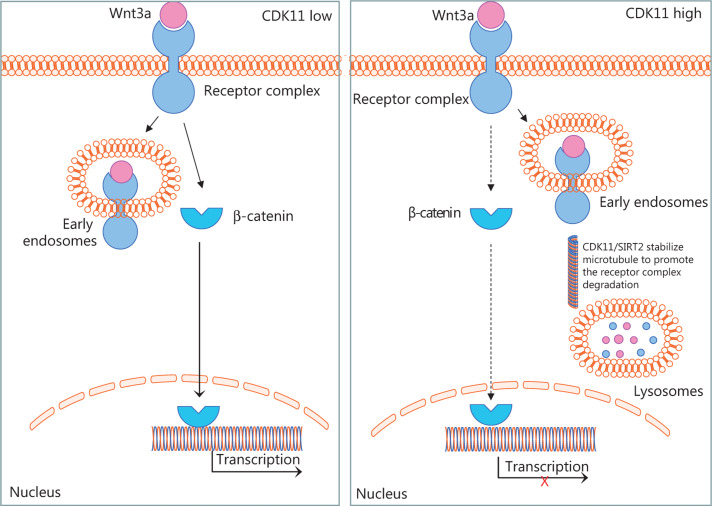
Schematic of CDK11 regulation of Wnt/β-catenin signaling, according to our results. The diagram shows that CDK11 regulates the trafficking of Wnt/β-catenin signaling receptor complexes between early endosomes and lysosomes by modulating microtubule stability. When CDK11 is present at a low level, the receptor complexes are retained in early endosomes, and Wnt/β-catenin signaling is active. When CDK11 is present at a high level, microtubule stability is enhanced, and the receptor complexes traffic from early endosomes to lysosomes for degradation increases, thus leading to the inactivation of Wnt/β-catenin signaling.

Intracellular trafficking of receptors between endosomes has been shown to be dependent on microtubules^49^, and microtubule stability is required for transport from early endosomes to late endosomes or lysosomes^41,42^. Whether active microtubule-based transport might play a role in Wnt/β-catenin signaling remains a matter of speculation. Here, we showed that CDK11 plays a role in the transport of Wnt/β-catenin signaling receptor complexes through the endolysosomal system, on the basis of the regulation of microtubule stability (**Figure 7**). Accumulation of receptor complexes in Wnt/β-catenin signaling after CDK11 depletion was not observed in SW480 cells, thus suggesting that intact APC may play an important role in the recruitment of Axin1 and GSK3β as a whole after Wnt3a binds Fzd and LRP6—a possibility requiring further research.

CDK11’s functions are particularly versatile among those of CDK family members. Previous studies have found that CDK11 is up-regulated in breast cancer^50^, multiple myeloma^51^, osteosarcoma^52^, and esophageal squamous cell carcinoma^53^, and down-regulation of CDK11 arrests growth and induces apoptosis in these cancer cells, thus suggesting that CDK11 acts as an oncogene. However, CDK11 has also been found to be depleted in several cancers, such as neuroblastoma^54^, melanoma^55^, and non-Hodgkin’s lymphoma^56^. These results imply that CDK11 may be a tumor suppressor gene. In this study, our data suggest that CDK11 may function as a tumor suppressor by deregulating Wnt/β-catenin signaling, at least in cervical cancer cells.

## Conclusions

In summary, we conducted a kinase RNAi screen to identify kinases regulating Wnt/β-catenin signaling, from which we identified CDK11 as a negative regulator. In the underlying regulatory mechanism, Wnt/β-catenin signaling receptor complexes are destabilized, and cellular trafficking of signal molecules to lysosomes for degradation is increased via CDK11 regulation. The details of the association of CDK11 with Wnt/β-catenin signaling in the endosome and lysosome trafficking machinery remain to be further elucidated.
